# Repurposing of drugs against methyltransferase as potential Zika virus therapies

**DOI:** 10.1038/s41598-023-33341-6

**Published:** 2023-05-15

**Authors:** Rohit Shukla, Anshuman Chandra, Anuj Kumar, Pallavi Kandpal, Himanshu Avashthi, Vijay Kumar Goel, Imteyaz Qamar, Nagendra Singh, David J. Kelvin, Tiratha Raj Singh

**Affiliations:** 1 Department of Biotechnology and Bioinformatics, Jaypee University of Information Technology (JUIT), Waknaghat, Solan, Himachal Pradesh 173234 India; 2 Centre for Excellence in Healthcare Technologies and Informatics (CEHTI), Jaypee University of Information Technology (JUIT), Waknaghat, Solan, Himachal Pradesh 173234 India; 3 School of Biotechnology, Gautam Buddha University, Gautam Buddh Nagar, Greater Noida, Uttar Pradesh 201312 India; 4 School of Physical Science, Jawaharlal Nehru University, New Delhi, 110067 India; 5 Laboratory of Immunity, Shantou University Medical College, Shantou, China; 6 Department of Microbiology and Immunology, IWK Health Center, Canadian Centre for Vaccinology CCfV, Faculty of Medicine, Dalhousie University, Halifax, Canada; 7 Department of Pediatrics, IWK Health Center, Canadian Centre for Vaccinology CCfV, Faculty of Medicine, Dalhousie University, Halifax, Canada; 8 European Virus Bioinformatics Center, Leutragraben 1, Jena, Germany; 9 ICMR-NIMR, Dwarka, New Delhi, 110077 India; 10 Division of Agricultural Bioinformatics, ICAR-Indian Agricultural Statistics Research Institute, Pusa, New Delhi, India

**Keywords:** Virtual drug screening, Computational biology and bioinformatics, Drug discovery

## Abstract

In recent years, the outbreak of infectious disease caused by Zika Virus (ZIKV) has posed a major threat to global public health, calling for the development of therapeutics to treat ZIKV disease. Several possible druggable targets involved in virus replication have been identified. In search of additional potential inhibitors, we screened 2895 FDA-approved compounds using Non-Structural Protein 5 (NS5) as a target utilizing virtual screening of in-silco methods. The top 28 compounds with the threshold of binding energy −7.2 kcal/mol value were selected and were cross-docked on the three-dimensional structure of NS5 using AutoDock Tools. Of the 2895 compounds screened, five compounds (Ceforanide, Squanavir, Amcinonide, Cefpiramide, and Olmesartan_Medoxomil) ranked highest based on filtering of having the least negative interactions with the NS5 and were selected for Molecular Dynamic Simulations (MDS) studies. Various parameters such as RMSD, RMSF, Rg, SASA, PCA and binding free energy were calculated to validate the binding of compounds to the target, ZIKV-NS5. The binding free energy was found to be −114.53, −182.01, −168.19, −91.16, −122.56, and −150.65 kJ mol^−1^ for NS5-SFG, NS5-Ceforanide, NS5-Squanavir, NS5-Amcinonide, NS5-Cefpiramide, and NS5-Ol_Me complexes respectively. The binding energy calculations suggested Cefpiramide and Olmesartan_Medoxomil (Ol_Me) as the most stable compounds for binding to NS5, indicating a strong rationale for their use as lead compounds for development of ZIKV inhibitors. As these drugs have been evaluated on pharmacokinetics and pharmacodynamics parameters only, in vitro and in vivo testing and their impact on Zika viral cell culture may suggest their clinical trials on ZIKV patients.

## Introduction

RNA viruses are molecular pathogens that cause diseases at a rapid pace, making them the fastest-growing type of pathogen. These viral pathogens show significant genetic variability and opt for distinct replication strategies to replicate within cells^1^. ZIKV is an arthropod-borne member of the Flaviviridae family that spreads through the bite of an infected Aedes mosquito species such as *Aedes africanus, A. luteocephalus, A. aegypti*, and *A. albopictus*. It was first discovered in parts of Africa in 1947, and its outbreak was seen in other geographic areas such as the Far East, Europe, North America, the Caribbean, and Central/South America^2^. ZIKV causes mild symptoms similar to those of other flavivirus-borne diseases, such as fever, muscular discomfort, headache, joint pain, exhaustion, and conjunctivitis. However, the 2015 outbreak in Egypt, the Pacific Islands, and Brazil drew the attention of the scientific community because of the high infection rate and its association with life-threatening diseases such as Neonatal Microcephaly (NM) and adult Guillain–Barre syndrome (GBS)^3^. A rise in cases of NM and GBS was observed during the ZIKV outbreak. NM is a condition in which a child's head development is hampered whereas GBS is an immune system related disease of the peripheral nervous system that is usually triggered by infection^4^. NM was observed in foetuses of pregnant women who tested positive for ZIKV during the first trimester of pregnancy. GBS causes weakness and loss of senses that may result in paralysis. It was reported that the male population is more prone to GBS than female^5,6^.

ZIKV is a positive-sense RNA genome with an open reading frame (ORF) containing structural protein at the 5’ end and non-structural proteins (NS) at the 3’ end^7^. The structural proteins are Capsid (C), preMembrane (Pre-M), and Envelope (E) proteins and all of them aid in mature virus particle formation^8^. The non-structural proteins NS (1, 2A, 2B, 3, 4A, 4B, 5) are responsible for host defense response evasion and genome replication^9^. ZIKV transmits through mosquito bites after which the virus attaches to the host receptor through a viral envelope protein. ZIKV internalization in the host occurs via apoptotic mimicry^10^. Following internalization, a fusion between the viral membrane and the endosomal membrane of the host occurs, releasing viral RNA into the cytoplasm for propagation^11^. Viral RNA is transcribed and translated forming a single polyprotein, in the order of Capsid-PreMembrane-Envelope-NS (1–2A–2B–3–4A–4B–5). Virus assembly occurs in the endoplasmic reticulum after successful transcription in the nucleus of the host by RNA-dependent RNA polymerase (RdRp) and is transported to the Golgi complex with virion buds. Pre-M protein is then cleaved further, resulting in viral particle maturation and exocytosis from the Golgi^6,12^. Non-structural proteins NS1, NS3, NS4B, and NS5 are known to inhibit the signaling pathways of the cell^12^. Activities of non-structural proteins such as NS2B are also well-known, which works as a cofactor and cleaves protein at dibasic sites in the cytoplasm^12^. NS5 plays a key role in viral propagation as it has RNA-dependent RNA polymerase (RDRP) activity and methyl transferase (MTase) for capping of nascent RNA^13^. Capping is a multi-step process that requires multiple enzymes. In the case of ZIKV, this multi-step process is carried out by a single enzyme, NS5. Two subsequent methylations are required to cap viral RNA. The first methylation process occurs when the type-0 cap structure (m7GpppN-RNA) is formed by (a) removal of phosphate from triphosphate of nascent RNA at the 5` end; (b) when GMP is attached to the diphosphate end to yield a cap core structure; and (c) when GMP is finally methylated to form the m7GpppN-RNA cap. The second methylation process forming the type-I cap structure (m7GpppNm-RNA) occurs by methylation of the first nucleotide of the triphosphate bridge^12,13^.

Capping of viral RNA has distinct functions: (a) guanosine cap of triphosphate prevents activation of the host immune response; (b) type-0 structure is important for viral replication; and (c) type-I structure helps in evading the host immune response by mimicking cellular mRNA^13^. The mechanism of capping nascent RNA via MTase is an important step for ZIKV infection^13^.

Drug designing is a time-consuming process. Most of the drug development time is taken in absorption, distribution, metabolism, elimination, and toxicity (ADMET) evaluation, which are critical parameters in drug safety analysis. Drug repurposing is an approach where scientists evaluate an FDA-approved drug against other diseases to remove the ADMET time barrier. Drug repurposing has been proven as a powerful approach to finding new therapies for SARS-CoV-2 during the pandemic^14–17^. Several FDA-approved drugs such as chloroquine, hydroxychloroquine, azithromycin, and ivermectin etc. were directly used to alter disease progression without any major clinical trials. Because these drugs were already tested against other diseases and very well evaluated in terms of their ADMET evaluation, patients received the clinical benefits from these drugs without any serious adverse effects^18^. Consequently, existing drugs can be tested against several diseases other than the ones for which that have already been approved. Many drugs have been evaluated against several COVID-19 drug targets and scientists have proposed potential compounds against several targets using the combinatorial chemistry approaches^19^. For example, Ceforanide is an antibacterial drug while saquinavir is an antiviral drug yet both these drugs proved efficacious against SARS-CoV-2 targets. Ceforanide is a well-known anti-bacterial compound that belongs to the semi-synthetic, broad-spectrum, beta-lactam, second-generation cephalosporin antibiotic family. It inhibits bacterial cell wall synthesis by inactivating penicillin binding proteins (PBPs) through the alteration of the final transpeptidation step which is necessary for peptidoglycan cross-linking, which is a major cell wall component^20^. In an in-silico study, various antiviral drugs have been tested against SARS-CoV2 where Ceforanide showed tight binding with a high number of hydrogen bonds against the RdRp viral protein^21^. It showed that it can be used as an antiviral compound and can be repurposed against ZIKV disease. Saquinavir is a well-known HIV-1 protease inhibitor developed in 1995; it works in combination with ritonavir against human immunodeficiency virus-1 (HIV-1) infection. Alone, Saquinavir showed very poor bioavailability (approximately 4%); however, when combined with ritonavir, which is a potent enzyme inhibitor that increases the serum concentration of Saquinavir, it performs with higher antiviral activity^22,23^. Recent studies showed that Saquinavir can inhibit the main protease (M-pro) of SARS-CoV-2^24,25^. It was also tested in cancer, where it performed well, and in the future it can also be used as an anti-cancer drug^26^. The above illustrate that Saquinavir can also halt ZIKV disease progression by inhibiting NS5. Likewise, in this study, we have chosen drugs already approved by the FDA for use against-other diseases to test them against ZIKV to reduce the time and cost involved in developing new anti-ZIKV compounds.

**Figure 1:**
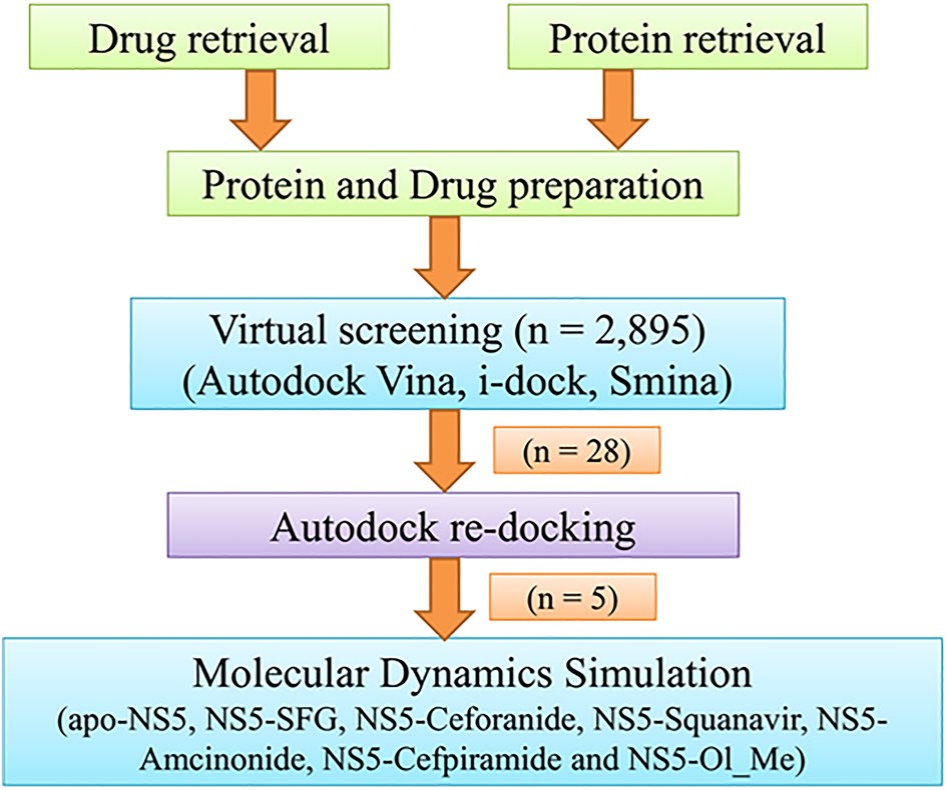
The brief methodology of the work.

Three-dimensional coordinates of ZIKV NS5 MTase (NS5) were retrieved from the Protein Data Bank. The structure was prepared for virtual screening and 2,895 FDA-approved compounds were screened against the NS5. Using cross-docking and molecular dynamic simulation (MDS) studies, Cefpiramide and Olmesartan_Medoxomil (Ol-Me), which are used to treat bacterial infection and hypertension, were selected as lead compounds. The brief methodology is shown in Fig. 1.

## Materials and methods

### Structure and ligand retrieval

**Figure 2:**
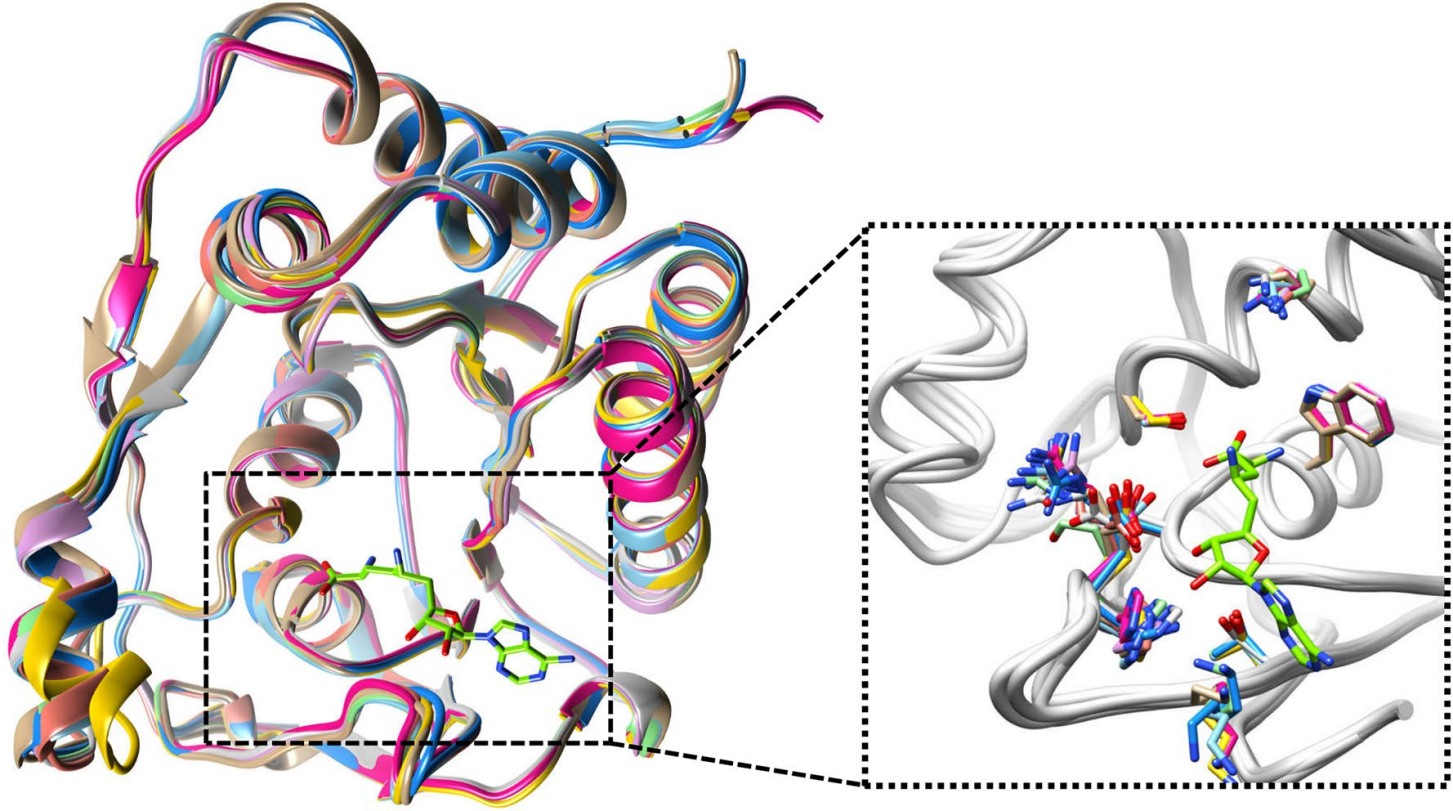
Superimposition of ZIKV methyltransferase with other structures. Inset shows the binding site residues orientation. This figure was generated with the help of UCSF Chimera v.1.13.2 (https://www.cgl.ucsf.edu/chimera/) software.

The protein 3D structure of the ZIKV, methyltransferase or NS5 (PDB ID: 5MRK, X-ray, 1.9 Å)^27^ was retrieved from the Protein Data Bank (PDB). It is co-complexed with an inhibitor, Sinefungin (SFG)^27^. We compared the ZIKV, methyltransferase (PDB ID: 5MRK) with other reported ZIKV methyltransferase structures and found that all the structures share similar folds. We observed that binding site residue orientation is also similar for all the structures. The RMSD between the 5MRK and 5GOZ, 5M5B, 5WZ1, 5NUJ, and 5NJV was 0.479, 0.362, 0.396, 0.349 and 0.390 Å respectively (Fig. 2). SFG is isolated from the Streptomyces species and it is a natural nucleoside related to S-adenosylmethionine. It has antiviral, antifungal, and antiparasitic activity. It is a well-known antifungal antibiotic and competes with S-adenosyl-1-methionine (SAM), the natural substrate of MTases^28^. SFG has shown intramolecular interactions with ZIKV methyltransferase^27^; therefore, we utilized SFG as a control compound for comparing binding of the predicted ligands. FDA-approved compounds (n = 2895) were retrieved from the ZINC database (https://zinc.docking.org/) in .*mol2* file format. ZINC is a large database and comprises millions of compounds in different subsets.

### Protein and ligand preparation

Heteroatoms such as Cl^-^, SFG, and water, etc. were removed from the NS5 protein structure using Chimera 1.13.2 and then the apo-protein structure was employed for energy minimization using Chimera 1.13.2^29^. We used Amber ff99SB force field^30^ and the 100 steepest descent steps were run for removing the steric clashes in the protein. The protein structures was converted into* .pdbqt* file format by using Autodock^31^ prior to the virtual screening. All the desired hydrogen atoms were added in the structure and the Kollmann charges were set. The ligands from ZINC database in *.mol2* format contains all the hydrogens with proper geometry; therefore, they were directly used for the .*pdbqt* conversion instead of energy minimization. The ligands were taken and converted using *prepare_ligand4.py* python script provided in the AutoDock Tools. This automated script adds all the charges, removes the steric clashes, and converts the *.mol2* file into *.pdbqt* file format. Then the protein and ligands (*.pdbqt* format) were used for the virtual screening through Autodock Vina v.1.2.0^32^ using the in-house developed pipeline (https://github.com/shuklarohit815/pyVSvina).

### High-throughput screening

High-throughput virtual screening (HTVS), a widely used technique to identify the potential inhibitors from a large dataset^33^, avoids the extensive effort of synthesizing individual molecules and the respective cost to identify the lead molecule. Due to invention of computational power, the HTVS technique is able to screen compound datasets within hours. We prepared a grid based on the co-crystallized ligand SFG. The SFG showed the interactions with Ser56, Gly86, Trp87, Lys105, His110, Glu111, Asp131, Val132, Asp146, and Ile147 in the co-crystallized structure^27^. We selected the same binding residues and created a grid (Center_X = 18, Center_Y = 6, Center_Z = 5 and Size_X = 20, Size_Y = 20 and Size_Z = 20) and utilized the same grid for the three docking software analyses. We next screened the FDA-approved compound dataset against the NS5 enzyme. The prepared protein and ligands were docked with the NS5 enzyme using Autodock Vina v.1.2.0^32^, I-dock v.0.1 (https://GitHub.com/HongjianLi/idock) and Smina software v.0.1 (https://sourceforge.net/projects/smina/). The compounds with the best affinity were selected for further analysis. The resulting 28 compounds were employed for redocking studies by using Autodock Tools (ADT).

### Molecular docking analysis

Cross-docking from multiple software is a widely used approach to remove false positive binders^34,35^. We selected 28 compounds from the virtual screening analysis based on their binding properties with the human receptors. These compounds were employed for cross-docking analysis using Autodock Tools (ADT). The Autodock uses the Lamarckian genetic algorithm (LGA) for producing the docking pose. The same grid box, which was set for virtual screening, was utilized for the docking. The grid box was set based on SFG binding residues to these coordinates (Center_X = 18, Center_Y = 6, Center_Z = 5 and Size_X = 20, Size_Y = 20 and Size_Z = 20). In the Autodock tools, the binding affinity was evaluated in the following two steps: first the energy was calculated in an unbound state followed by energy calculation of the NS5-ligand complexes. The difference between the first and second steps was then evaluated. The sum of the freedom of torsional degree was used for the calculation of conformational entropy. The free energy of binding was calculated as:

1 $$\Delta G = \left( {V_{bound}^{L - L} - V_{unbound}^{L - L} } \right) + \left( {V_{bound}^{P - P} - V_{unbound}^{P - P} } \right) + \left( {V_{bound}^{P - L} - V_{unbound}^{P - L} + \Delta S_{conf} } \right)$$

where the protein and ligand are referred to as *P* and* L*, pairwise evaluation is denoted by* V*, and *ΔS*_*conf*_ denotes the loss of conformational entropy during binding. One hundred binding poses for each ligand were generated by using the Lamarckian genetic algorithm. The complexes were selected through the binding mode and binding affinity analysis. Finally, the top five compounds were selected based on their interaction and binding energy and the control ligand in complex form were employed for MDS.

### Molecular dynamics simulation

MDS is a widely used technique for predicting protein–ligand complex stability. We performed 150 ns MDS for evaluating the stability of the protein–ligand complexes. We created seven systems (one for apo-NS5, one for NS5-SFG, and the other 5 for the selected hits) and employed them for MDS studies. All the systems were placed in a cubic box and water molecules were filled using the gmx *solvate* tool. Ions were added for the neutralization of the systems. The neutralized systems were employed for energy minimization using the steepest descent algorithm for 5000 steps for removing the steric clashes and irregular geometry. After that Constant Number of particles, Volume, and Temperature (NVT) and Constant Number of particles, Pressure, and Temperature (NPT) simulation of 1 ns each was performed for maintaining the temperature, pressure, and volume of the systems. The van der Waals and short-range electrostatic interactions applied a cutoff of 1.0 nm. The Particle Mesh Ewald method was used for treating the large range electrostatic interaction^36^. The LINCS algorithm was used for constraining all the bonds^37^. Finally, all the systems were employed for 150 ns MDS studies and the coordinates were saved for every 0.2 fs. Analyses of RMSD, RMSF, Rg, SASA, intramolecular interaction, PCA, and MM-PBSA were performed with *gmx rms, gmx rmsf, gmx gyration,* and *gmx sasa, etc.* tools respectively as earlier described^38,39^. The trajectory was visually analyzed using Chimera 1.13.2^29^.

### Principal component analysis

The principal component analysis (PCA) is a widely used method to identify the correlated motions which are induced by ligand binding^40^. The conformational ensemble of MD simulation was used to conduct PCA analysis. A covariance matrix was constructed on the basis of Cα atom displacement^41,42^. The inbuilt tool of Gromacs *gmx covar* and *gmx anaeig* was used for the eigenvector and eigenvalue generation. The covariance matrix (*C*_*ij*_) is defined from the following equation:

2 $$C_{ij} = (r_{i} - < r_{i} > ) \times (r_{j} - < r_{j} > )$$

where, the *r*_*i*_ and *r*_*j*_ are the mass-weighted Cartesian coordinates of the *i*_*th*_ and *j*_*th*_ Cα atoms.

The eigenvalues and their respective eigenvectors were obtained by the diagonalization of the covariance matrix. Finally, the plots were drawn by the Origin 6.0 software. We also took the first two Principal Components (PCs) and plotted them against each other to generate the 2D projection plot. This plot was used for the three-dimension free energy landscape analysis as described earlier^43^.

### Binding free energy calculations

The binding free energy for all the complexes was calculated using the molecular mechanics Poisson-Boltzmann surface area (MM-PBSA) tool^44^. The final 500 snapshots generated from each trajectory was used for the analysis. The binding free energy was calculated using this equation:

$$\Delta G_{bind} = \, \Delta G_{mm} + \, \Delta G_{sol} {-} \, T\Delta S$$

The binding affinity *ΔG*_*mm*_ (molecular mechanics energy) was estimated considering the electrostatic and van der waals interactions. The polar and non-polar contributions estimate the *ΔG*_*sol*_ (solvation free energy). The solvent accessible surface area (SASA) describes the nonpolar solvation free energy. The entropy (TΔS) was excluded due to its high computational cost.

## Results

### Virtual screening

Structure-based virtual screening was conducted for the identification of novel inhibitors against NS5 (PDB ID: 5MRK). We utilized Autodock Vina, Smina, and I-dock for this purpose. From all these tools we observed that ZINC03830383 (Carbinoxamine) was the highest energy compound based on analyses of all three docking software. A binding affinity of −11.2 kcal/mol, −11.42 kcal/mol, and −12.9 kcal/mol were obtained from Autodock Vina, I-dock, and Smina for ZINC03830383. ZINC08034121 showed that the least binding binding affinity from all the docking software, which was −2.5, −2.39, and −2.6 kcal/mol from Autodock Vina, I-dock, and Smina. The binding affinity of 2895 compounds is shown in Supplementary Table 1. From this screening we selected compounds based on their binding energy as well as their interaction analysis. Further selected compounds were used for the cross-docking analysis using Autodock Tools.

### Docking result analysis

Based on the virtual screening results, we selected the 28 best compounds for the cross-docking studies using Autodock. ZINC3914596 (Saquinavir) showed the highest binding affinity of −9.77 kcal/mol, while ZINC3938482 (Posaconazole) showed the best binding energy of −6.03 kcal/mol. All 28 compounds showed a favorable binding affinity toward NS5, indicating that they can act as inhibitors. From this result we selected five compounds that showed good binding affinity from the three docking software which do not directly target human receptors except Ol_Me; however, they showed binding to the bacterial or viral receptors^45–47^. The details of binding energy, hydrogen bond interactions, and other interacting residues are shown in Supplementary Table 2. The ZINC ID, drug name, and binding affinity of selected 5 compounds with the control compound are shown in Table 1.

**Table 1:** 2D structure and binding energy from all the softwares for the selected drugs.

ZINC ID	Drug name	2D structure	AutoDock	AutoDock Vina	idock	Smina
ZINC04217451	SFG		−7.2	−7.7	−7.76	−8.1
ZINC3830435	Ceforanide		−9.54	−9.2	−9.09	9.2
ZINC3914596	Saquinavir		−9.77	−9.5	−9.31	−9.6
ZINC3977777	Amcinonide		−9.52	−10.0	−10.7	−10.07
ZINC4215257	Cefpiramide		−9.48	−10.1	−9.10	−10.2
ZINC14294279	Olmesartan Medoxomil		−9.1	−9.0	−9.14	−9.2

The binding affinity is in Kcal/mol for all the softwares.

#### NS5–SFG interaction

**Figure 3:**
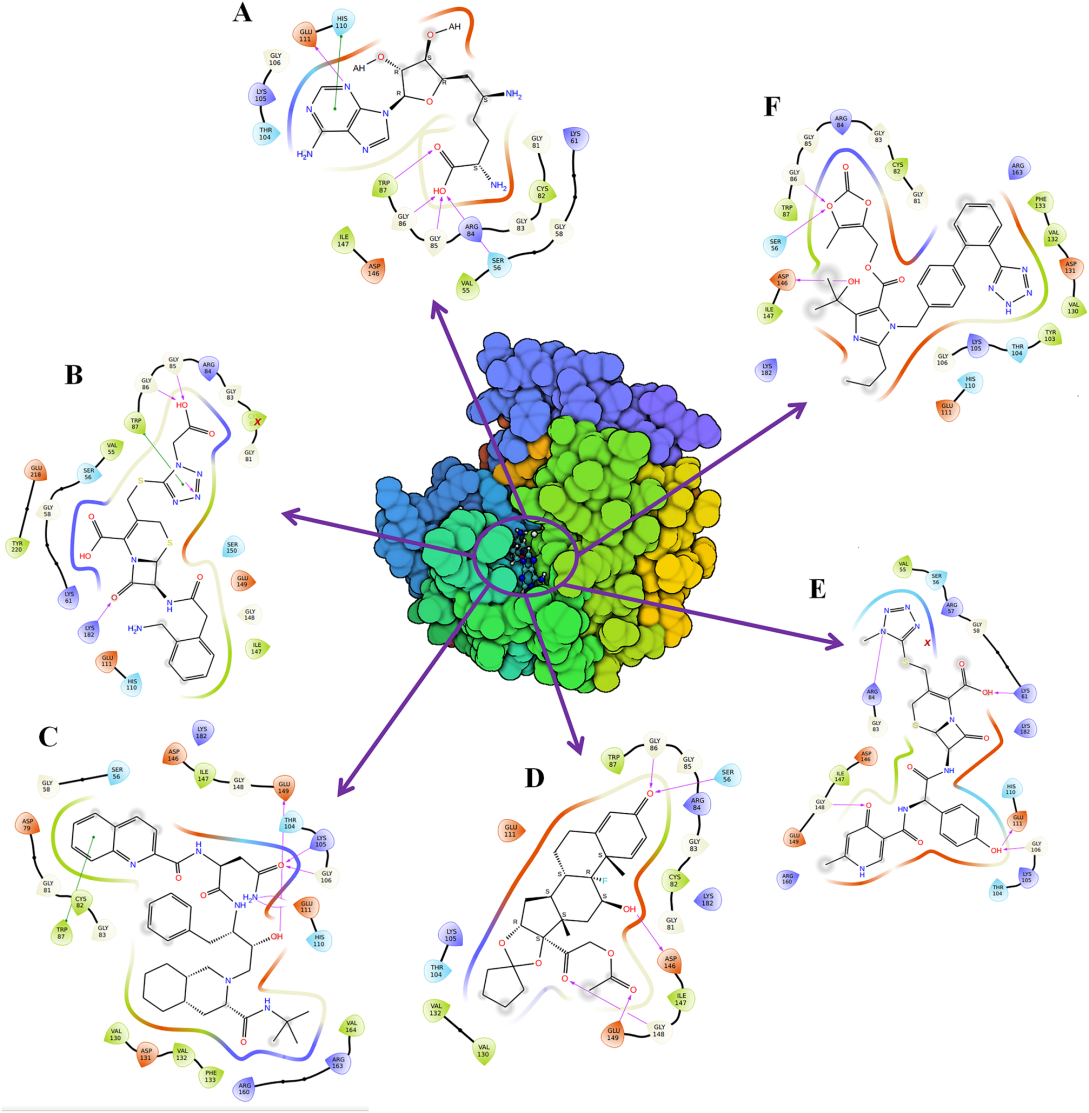
Binding of drugs to NS5. **(A)** NS5–SFG, **(B)** NS5–Ceforanide, **(C)** NS5–Squanavir, **(D)** NS5–Amcinonide, **(E)** NS5–Cefpiramide, and **(F)** NS5–Olmesartan_Medoxomil. Amino acids interacting with the drugs are represented as negatively charged (orange), positively charged (blue), polar (cyan), and glycine (white), respectively. Binding modes of the drugs were rendered with the help of academic version of Maestro (https://www.schrodinger.com/products/maestro) and PyMOL 2.4 (https://pymol.org/2/) packages.

SFG was used as the positive control and demonstrated binding affinities of −7.2, −7.7, −7.76, and −8.1 kcal/mol for Autodock, Autodock Vina, idock, and Smina analyses respectively. The NS5-SFG complex was stabilized by various forces including hydrophobic, hydrogen bond, and electrostatic interactions. SFG formed hydrogen bonds with Ser56, Gly85, Gly86, Trp87, and Glu111 residues in NS5. The Interaction details of SFG are shown in Fig. 3A. The residue details are given in Supplementary Table 2.

#### NS5–Ceforanide interaction

Ceforanide showed binding affinities of −9.54, −9.2, −9.09, and −9.2 kcal/mol from Autodock, Autodock Vina, I-dock, and Smina respectively. The interaction details are shown in Fig. 3B from Autodock. The Autodock complex was stabilized by various interactions including hydrogen bond interactions, hydrophobic interactions, and ionic interactions. The Ceforanide complex was mainly stabilized by hydrogen bonds with Ser56, Lys61, Gly85, Trp87, and Lys182 of NS5. The interaction details are given in Supplementary Table 2.

#### NS5–Saquinavir interactions

The binding affinities for Saquinavir toward the NS5 receptor were calculated to be −9.77, −9.5, −9.31, and −9.6 kcal/mol based on Autodock, Autodock Vina, I-dock, and Smina respectively. Figure 3C illustrates the brief interaction of ligand with the NS5 protein. The complex was stabilized by various interactions including hydrogen bond interactions, hydrophobic interactions, and ionic interactions. Saquinavir forms hydrogen bonds with the NS5 residues including Lys105, Gly106, Glu111, and Glu149. The interaction details are given in Supplementary Table 2.

#### NS5–Amcinonide interaction

Amcinonide showed binding affinities of −9.52, −10.0, −10.7, and −10.07 kcal/mol from Autodock, Autodock Vina, I-dock, and Smina respectively. We analyzed the binding of the drug using Autodock (Fig. 3D**)**. The complex was stabilized by hydrogen bonds and hydrophobic interactions. Amcinonide formed hydrogen bonds with NS5 residues including Ser56, Gly86, Gly148, and Glu149 residues of NS5 (Supplementary Table 2).

#### NS5–Cefpiramide interactions

Cefpiramide showed binding affinities of −9.48, −10.1, −9.10, and −10.2 kcal/mol from Autodock, Autodock Vina, I-dock, and Smina respectively. The Autodock complex interaction details are shown in Fig. 3E. The complex was stabilized by hydrogen bonds with NS5 residues including Lys61, Arg84, Gly106, and Gly148. Various other interactions are shown in Supplementary Table 2.

#### NS5–Ol_Me interaction

The binding energy for Ol_Me was −9.1, −9.0, −9.14, and −9.2 kcal/mol from Autodock, Autodock Vina, I-dock, and Smina, respectively. The interaction diagram of Ol_Me and NS5 is shown in Fig. 3F. The drug-receptor complex was stabilized by various interactions including hydrogen bond interactions, hydrophobic interactions, and ionic interactions. Ol_Me formed hydrogen bonds with NS5 residues including Ser56 and Gly86 residues. The interaction details are given in Supplementary Table 2.

### Molecular dynamics simulation

MDS is a widely used technique to evaluate protein–ligand complex stability. It mimics natural conditions and determines the dynamics of the predicted complex. We employed five predicted complexes for 150 ns with SFG as the control compound along with apo-NS5. Finally, seven systems were created and simulated to achieve the protein–ligand dynamics.

#### Stability analysis

**Figure 4:**
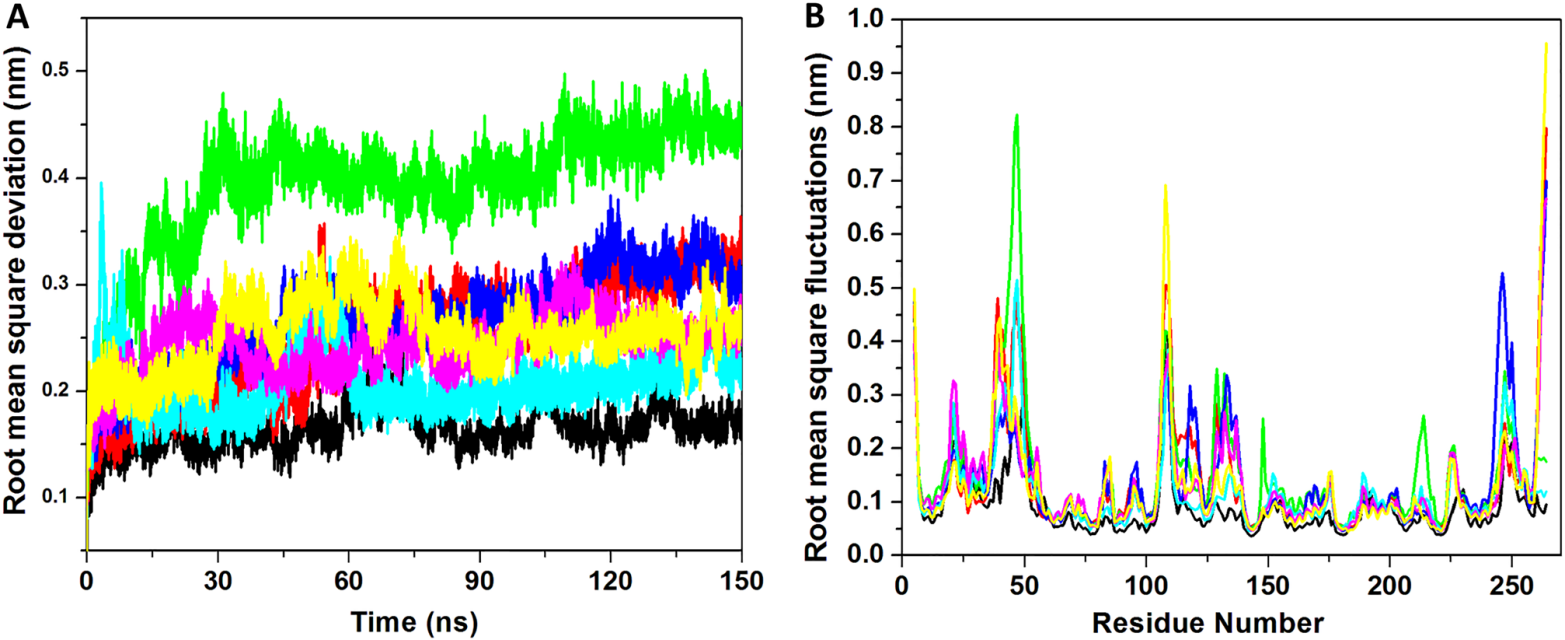
MDS studies using 150 ns. RMSD and RMSF for drugs binding to NS5. (**A**) RMSD for all the systems for 150 ns at 300 K. (**B**) RMSF for all the residues. The apo-NS5 (black) and NS5 complex with SFG (red), Ceforanide (green), Saquinavir (blue), Amcinonide (cyan), Cefpiramide (pink), and Ol_Me (yellow) are represented.

The stability of all the systems was analyzed by root mean square deviation (RMSD). The average values of all the complexes, apo-NS5, NS5–SFG, NS5–Ceforanide, NS5–Squanavir, NS5–Amcinonide, NS5–Cefpiramide, and NS5–Ol_Me, were 0.16, 0.25, 0.39, 0.26, 0.20, 0.24, and 0.25 nm respectively. The results are depicted in Fig. 4A. In the average value, the apo-NS5 showed a very low RMSD value, while the ligand binding inducing the conformational changes yielded high RMSD values, possibly resulting in high differentials between apo-NS5 and other complexes. The complex NS5–Amcinonide showed the least RMSD of 0.20 nm as compared to all other complexes. Figure 4A illustrates that all the trajectories showed a RMSD value of 0.2 to 0.3 nm while the NS5–Ceforanide complex showed a different RMSD pattern with values between 0.4 and 0.5 nm. These results suggest that all the complexes achieved stability and showed similar RMSD values as compared to the control compound.

#### Fluctuation analysis

The Root mean square fluctuation (RMSF) is a key important parameter to understand the conformational dynamics of the system during ligand binding. We calculated the RMSF of all the complexes and the results are shown in Fig. 4B. The complexes showed an average RMSF between 0.05 and 0.3 nm respectively. The average RMSF values for apo-NS5, NS5–SFG, NS5–Ceforanide, NS5–Squanavir, NS5–Amcinonide, NS5–Cefpiramide, and NS5–Ol_Me were 0.09, 0.13, 0.15, 0.13, 0.11, 0.13, and 0.12 nm respectively. Apo-NS5 showed the least RMSF value as compared to all other complexes. A few regions of sequence showed very high RMSF values as compared to other regions. The regions of amino acids in NS5 including 18–25, 36–52, 105–111, and 242–252, and the C-terminal region of the receptor showed the highest values. The 105–111 region is a flexible loop involved in forming the binding site of SFG and its flexibility may help in accommodating larger molecules; the fluctuation in this region indicated that the inhibitor binding alters conformation of the binding site. The overall results of RMSF indicates these complexes can alter the binding site, suggesting an induced fit mechanism of ligand binding. The RMSF results also suggest that all the complexes are relatively stable except NS5–Ceforanide.

#### Compactness analysis

**Figure 5:**
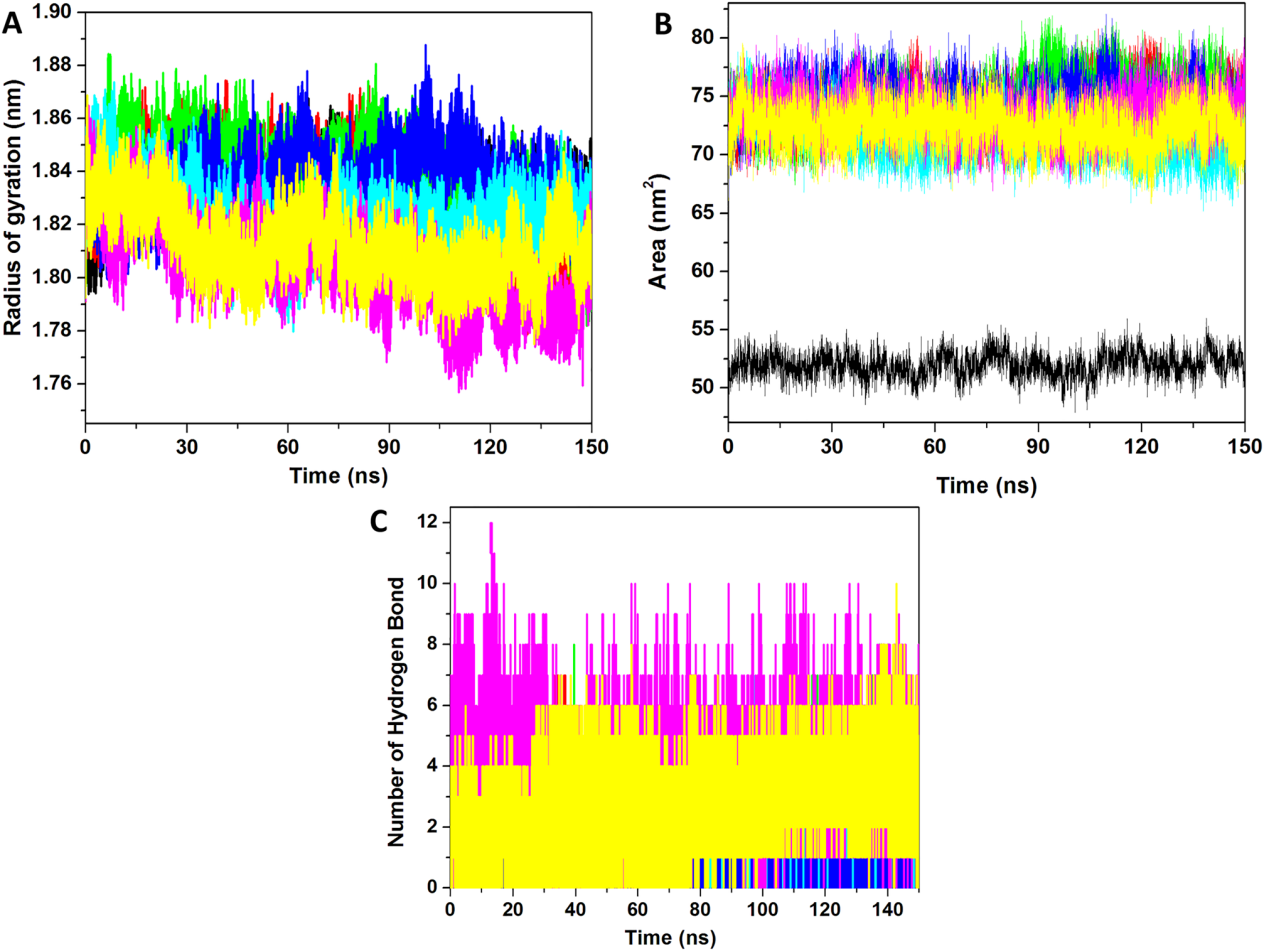
Stability analysis of drugs and NS5 complexes. (**A**) Rg of all the systems for 150 ns. (**B**) SASA value for all the systems. (**C**) Number of hydrogen bonds *vs.* time for all the ligands including control ligand. The apo-NS5 (black) and NS5 complex with SFG (red), Ceforanide (green), Saquinavir (blue), Amcinonide (cyan), Cefpiramide (pink), and Ol_Me (yellow) are represented.

The compactness of the protein is described by the radius of gyration (*Rg*) parameter and can be calculated by measuring the influence on the compactness of protein during ligand binding. We calculated the *Rg* values for measuring the conformational changes as shown in Fig. 5A. It is evident that NS5–Amcinonide, NS5–Cefpiramide, and NS5–Ol_Me showed lower Rg values compared to other complexes. The average *Rg* values for apo-NS5, NS5–SFG, NS5–Ceforanide, NS5–Squanavir, NS5–Amcinonide, NS5–Cefpiramide, and NS5–Ol_Me were 1.83, 1.82, 1.83, 1.83, 1.82, 1.80, and 1.81 nm respectively. These results showed that ligand binding induces compactness in the protein. The control compound NS5–SFG showed 1.83 nm and this value is similar for NS5–Amcinonide, while it is higher than those for NS5–Cefpiramide and NS5–Ol_Me. From this analysis, NS5–Amcinonide, NS5–Cefpiramide, and NS5–Ol_Me are more prominent in the terms of compactness analysis and this result is supported by RMSD and RMSF analyses.

#### SASA analysis

The solvent accessible surface area (SASA) defines the area which can occupy the solvent and plays a pivotal role in the maintenance of protein stability and protein folding. We calculated the SASA plot and the results are shown in Fig. 5B. The plot clearly demonstrates the lower SASA value for apo-NS5, while higher SASA values were obtained for the ligands. From the plot it is observed that all the predicted hits showed a similar SASA value; hence we next calculated the average value for all the trajectories. The average values for apo-NS5, NS5–SFG, NS5–Ceforanide, NS5–Squanavir, NS5–Amcinonide, NS5–Cefpiramide, and NS5–Ol_Me were 51.9, 73.9, 74.4, 74.4, 71.8, 73.4 and 72.4 nm^2^ respectively. Apo-NS5 showed the lowest SASA value, while ligand binding induced conformational changes and increased the SASA values in all ligand-NS5 SASA values. The three complexes, NS5–Amcinonide, NS5–Cefpiramide, and NS5–Ol_Me, showed lower values than the control compound.

#### Interaction analysis

NS5-ligand interactions are key to stabilization of the complex. Hydrogen bonds are very specific and play a pivotal role in the stability of the protein–ligand complex. We calculated the number of hydrogen bonds for the MD trajectory of NS5–ligand complexes (Fig. 5C**)**. The average number of hydrogen bonds for NS5–SFG, NS5–Ceforanide, NS5–Squanavir, NS5–Amcinonide, NS5–Cefpiramide, and NS5–Ol_Me was 7, 8, 6, 7, 12, and 10 respectively. The results illustrate that most of the complexes showed a higher number of hydrogen bonds compared to the number of hydrogen bonds of the control ligand (SFG) NS5 complex, with the single exception of the NS5–Squanavir complex. The NS5–Cefpiramide and NS5–Ol_Me showed multiple numbers of hydrogen bonds, 10 and 12 respectively, compared to the other selected ligands. The overall result suggests that these two complexes are more stable than other predicted hits.

#### Principal component analysis

**Figure 6:**
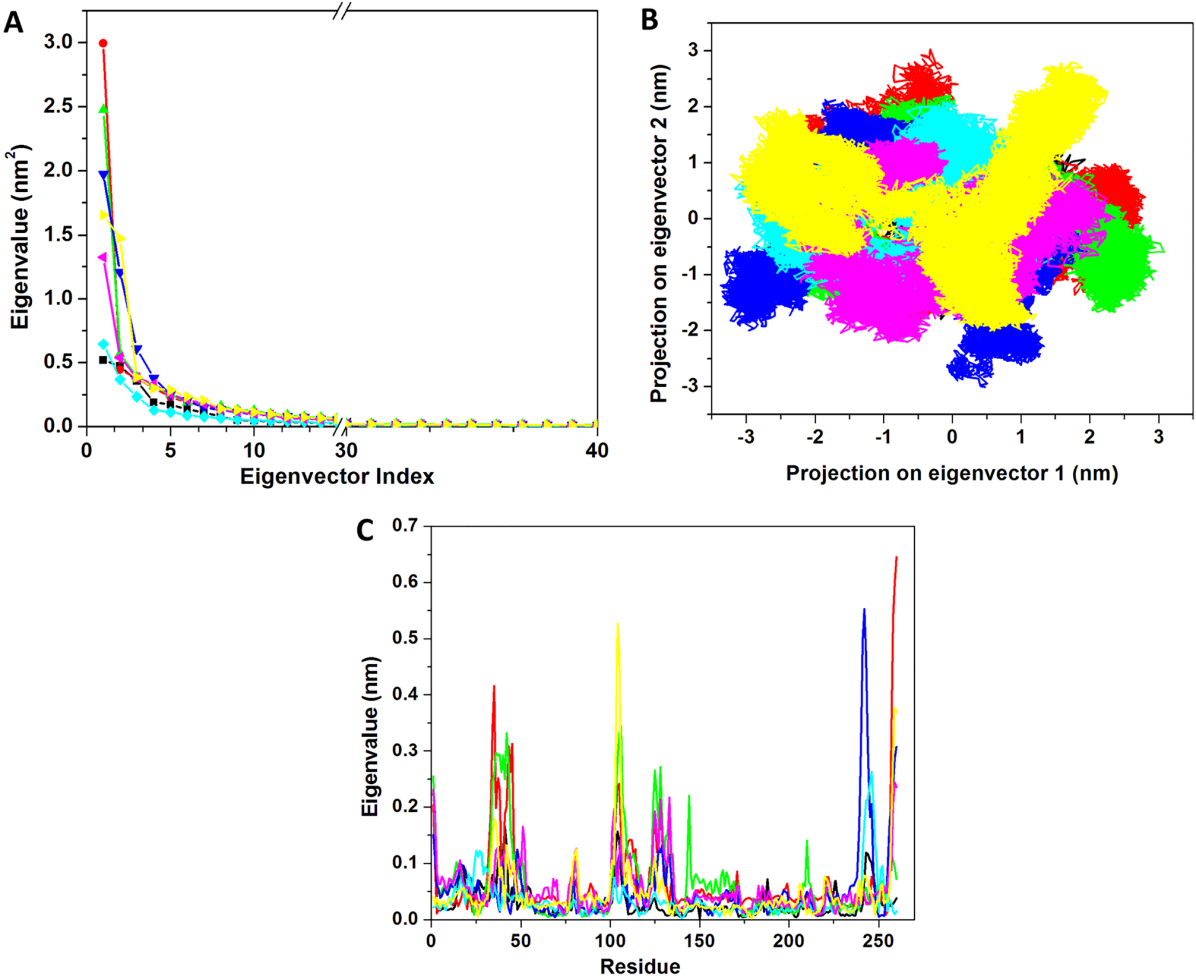
Principal component analysis. (**A**) Eigenvalue for the first 50 eigenvectors. (**B**) 2D projection motions for all the systems. (**C**) EigenRMSF for all the residues for the PC1. The apo-NS5 (black) and NS5 complex with SFG (red), Ceforanide (green), Saquinavir (blue), Amcinonide (cyan), Cefpiramide (pink), and Ol_Me (yellow) are represented.

We next predicted the correlated motions of NS5-drug complexes using PCA. All the NS5 bound complexes along with the apo-NS5 were analyzed using *gmx covar* and *gmx anaeig* tools. The eigenvalues were obtained by the diagonalization of the covariance matrix of atomic fluctuations. They are plotted in Fig. 6A. The first few eigenvectors play a key role in the overall dynamics of the system. We selected the first 50 eigenvectors which showed the motions of 79.33%, 80.82%, 78.14, 81.40%, 72.44%, 76.53%, and 80.29% for apo-NS5, NS5-SFG, NS5-Ceforanide, NS5-Squanavir, NS5-Amcinonide, NS5-Cefpiramide, and NS5-Ol_Me respectively. Figure 6A clearly shows that apo-NS5 has lower motions while the highest motion was observed in the NS5-Squanavir complex. Compared to the control compound, the predicted ligands showed lesser motion. It is evident that NS5-Ceforanide, NS5-Amcinonide, and NS5-Cefpiramide induced less motion and showed higher stability in the complex.

We selected the first two principal components and plotted them against each other (Fig. 6B**).** Apo-NS5 showed a very dense and stable cluster as compared to the control compound and other ligands. The control complex, NS5–SFG, showed a very large and unstable cluster while the predicted drugs also showed dense and stable clusters. The cluster of the predicted hits, NS5-Amcinonide, NS5–Cefpiramide, and NS5–Ol_Me, were more stable as compared to the other complexes.

After analysis we selected the first eigenvector to predict the correlated motions on the basis of residues. All the values were calculated based on eigenvalues *vs.* residues (Fig. 6C**)**. The average values for the apo-NS5, NS5–SFG, NS5–Ceforanide, NS5–Squanavir, NS5–Amcinonide, NS5–Cefpiramide, and NS5–Ol_Me were 0.03, 0.06, 0.06, 0.05, 0.03, 0.05, and 0.04 nm respectively. The values demonstrate that apo-NS5 showed little fluctuation while ligand binding to NS5 can induce fluctuation in the protein. The overall result suggests that NS5–Squanavir, NS5–Amcinonide, NS5–Cefpiramide, and NS5–Ol_Me are relatively more stable with minimal structural fluctuations.

#### Gibbs free energy landscape analysis

**Figure 7:**
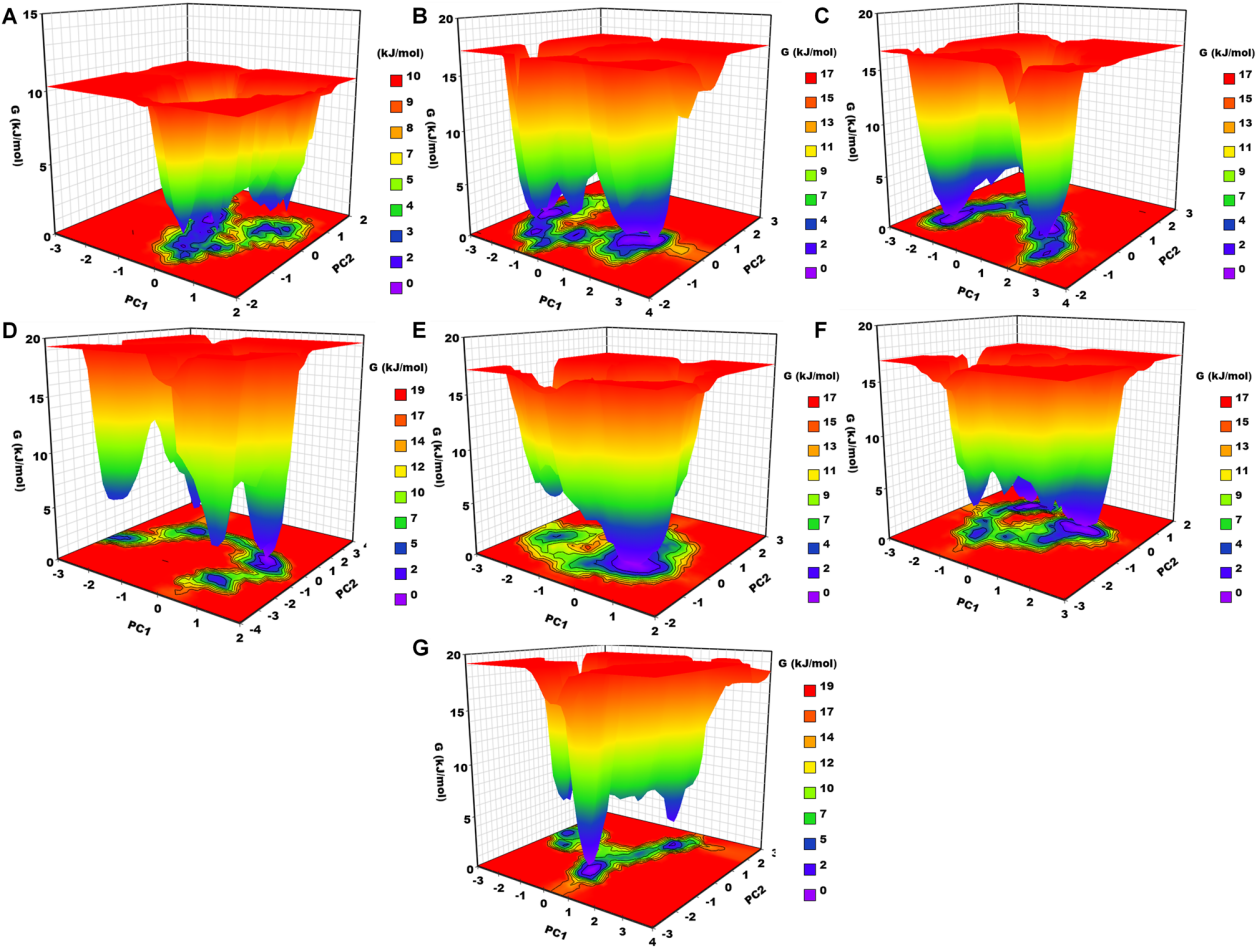
Gibbs free energy landscape. (**A**) apo-NS5, (**B**) NS5–SFG, (**C**) NS5–Ceforanide, (**D**) NS5–Saquinavir, (**E**) NS5–Amcinonide, (**F**) NS5–Cefpiramide and (**G**) NS5–Ol_Me.

The Gibbs free energy landscape analysis was performed using the first two principal components to get the free energy pattern of the apo-NS5, NS5–SFG, and predicted hits (Fig. 7). The apo-NS5 shows two energy funnels separated by energy barriers. The energy funnels are separated by various small energy minima and occupy the most space; the large area with intense blue color represents the stable cluster. The NS5–SFG also shows an apo-NS5-like pattern but here one energy funnel clearly represents a deep well and the other is bifurcated into four small energy minima, illustrating that the NS5–SFG complex changes through many conformational states to reach a stable state. The NS5–Ceforanide clearly displays two deep wells, with one well occupying area, as seen with the deep blue color. It indicates that this complex has two thermodynamically conformational states. The NS5–Saquinavir shows four clear deep wells which are separated by a high-energy barrier, symbolizing the four conformational states for this complex. The NS5–Amcinonide shows a single energy funnel with a deep blue color. It represents that this complex has only one conformation, indicating that it is the most stable complex as compared to the others. The NS5–Cefpiramide shows five energy funnels and they are very close to each other, forming a stable cluster. They are not separated by any high-energy barrier, again representing that this complex is also stable. The NS5–Ol_Me displays three deep wells or energy funnels, two of which show small conformational states with light blue color that are not thermodynamically favorable, while larger area with deep blue color illustrates the stable conformation of this complex. From the overall FEL results, we have concluded that the predicted hits undergo several transition states to reach the stable states and show the NS5–SFG like pattern.

#### Binding free energy analysis

The binding free energy describes the stability of the protein ligand complex. Binding energies were calculated for the last 500 snapshots for all NS5-ligand complexes (Table 2). The binding free energy for NS5–SFG, NS5–Ceforanide, NS5–Squanavir, NS5–Amcinonide, NS5–Cefpiramide, and NS5–Ol_Me were calculated as −114.53, −182.01, −168.19, −91.16, −122.56, and −150.65 kJ mol^−1^, respectively. The overall binding energy was evaluated including van der Walls energy, electrostatic energy, nonpolar solvation energy, and polar solvation energy. The highest binding affinity was observed for the NS5–Ceforanide complex while NS5–Amcinonide showed the least binding affinity. Cefpiramide and Ol_Me showed greater binding affinities than the control compound SFG–NS5 complex.

**Table 2:** Table represents the Van der Waals, electrostatic, polar solvation, SASA and binding energy in kJ·mol^−1^ for control compound and predicted hits.

Sr. No.	Compound name	Van der Waals energy	Electrostatic energy	Polar solvation energy	SASA energy	Binding energy kJ mol^−1^
1	SFG	−187.57 ± 8.94	−28.79 ± 9.27	121.90 ± 11.71	−20.08 ± 0.84	−114.53 ± 11.27
2	Ceforanide	−262.58 ± 12.28	−123.46 ± 12.66	226.42 ± 15.98	−22.4 ± 0.92	−182.01 ± 11.88
3	Squanavir	−219.51 ± 12.77	−24.88 ± 11.21	100.02 ± 20.80	−23.82 ± 1.56	−168.19 ± 13.57
4	Amcinonide	−172.15 ± 10.06	−64.83 ± 12.59	163.90 ± 21.07	−18.08 ± 1.22	−91.16 ± 15.02
5	Cefpiramide	−282.00 ± 13.71	−234.64 ± 29.50	420.028 ± 40.96	−25.95 ± 1.12	−122.56 ± 25.74
6	Olmesartan Medoxomil	−237.98 ± 14.84	−59.04 ± 9.95	167.70 ± 19.18	−21.32 ± 1.22	−150.65 ± 16.31

**Figure 8:**
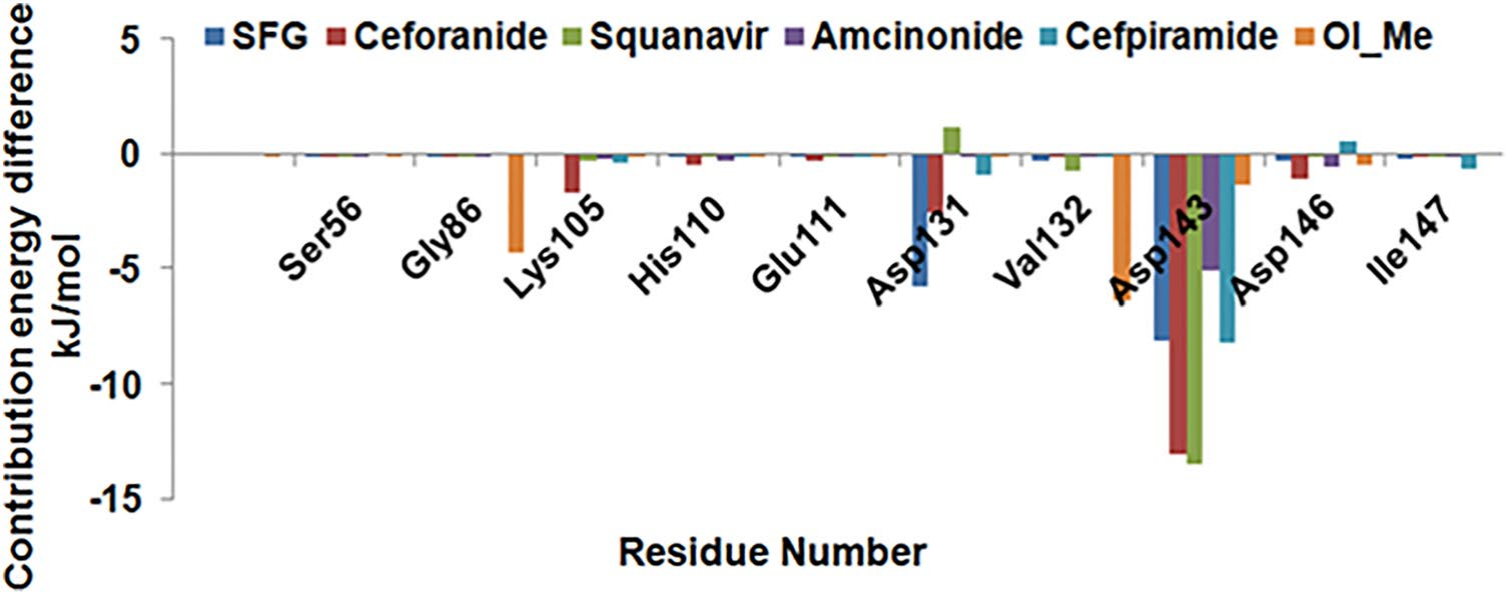
Binding free energy decomposition based on residues for all drug–NS5 complexes.

To evaluate the hotspot residues important for ligand stabilization, we calculated the per residue energy contribution (Fig. 8). To reduce the complexity of data for representation, we selected a few key binding site residues for the analysis**.** We observed that Lys105, Asp131, Asp143, and Asp146 residues play a crucial role in drug binding.

## Discussion

At present, various promising drug targets for ZIKV have been identified which can inhibit the viral growth. The anti-ZIKV compounds can reduce or halt the progression of ZIKV infection through the direct inhibition of key enzymes responsible for maintaining the biological function of ZIKV. Currently, many anti-ZIKV compounds are available which can inhibit various enzymes such as Temoporfin (NS2B/NS3 protease inhibitor)^48^, Suramin (NS3 helicase inhibitor)^49^, Nanchangmycin (envelope glycoprotein inhibitor)^50^, Sofosbuvir (NS5 RdRp inhibitor)^51^, and SFG (NS5 methyltransferase inhibitor)^13^. Although several approaches to developing new therapies have been tested over many years in the anti-ZIKV drug discovery program, no medications are available in the market against ZIKV. The compounds mentioned above are either cytotoxic or require high doses to inhibit the targets, which is a main reason that they have not been proposed as drugs in the market^52^. However, several targets are available for anti-ZIKV inhibitor identification with ZIKV NS5 as one of the major and most promising targets to date. The ZIKV NS5 crystal structure showed that the SAM binding site domain, called the SAM-dependent domain, could be a promising drug target^53^. Various structures complexed with the inhibitors targeting the SAM-dependent domain are available in the protein data bank. An analogue compound of SAM called SFG has been shown to inhibit the Dengue virus (DENV-2) and West Nile virus (WNV) with values of 0.63 mM and 14 mM IC_50_, respectively^54^. Various structures are available complexed with the SFG. Another SAM analogue called NSC 12155, identified in a virtual screening study, showed in vitro inhibitory activity against the WNV and Yellow fever virus (YFV) NS5 with an IC_50_ ranging from 0.50 to 3.00 mM. NSC 12155 showed an EC_50_ ranging from 1.00 to 7.00 mM against WNV, DENV-2 and Japanese encephalitis (JEV) in a cell-based assay^55^; However, NSC 12155 did not exhibit any activity against ZIKV. Theaflavin, a main component of tea, was also tested against NS5 of ZIKV, and showed 10.10 µM IC_50_ and 8.19 µM EC_50_ values against NS5. The authors also employed a computational study and predicted that theaflavin would show strong binding with D146 residue, which is a key catalytic residue^56^.

Drug repurposing is an effective approach to find potential drugs against many diseases, including ZIKV. It involves identifying drugs previously approved by the FDA for various diseases for use against other diseases for which they were not intended. For example, chloroquine, a widely used antimalarial drug, can significantly inhibit in vitro ZIKV infection with a value of 1–5 µM EC_50_^57,58^. Chloroquine alters the low pH-induced conformational changes which are necessary for fusion of the envelope protein with the endosomal membrane^59^; hence early stage ZIKV replication is blocked by chloroquine^60^. It is generally known that RNA viruses can mutate quickly; therefore, the identification of potential compounds with different mechanisms is required. Due to the importance of ZIKV NS5, we selected this enzyme as a drug target in this study and used computational approaches to find a novel drug through the drug repurposing approach. Computational approaches are practical^61^ as they avoid the time-consuming and expensive hurdles posed by ADMET in traditional drug designing^62^. Therefore, in this study, we retrieved the FDA-approved compound and screened it against NS5. We employed SFG as a control compound because it is co-crystallized with the crystal structure and has also shown good inhibitory activity against NS5 in several other studies. Virtual screening revealed various high-energy compounds; however, we selected only the five best antibacterial and antiviral compounds against NS5. The binding free energy and various structural parameters were calculated using the MDS trajectory, which revealed that Cefpiramide and Ol_Me can act as anti-ZIKV compounds with good binding affinity as compared to SFG. The drugs showed binding with key catalytic residue such as D146 which are required to perform the native enzyme activity^27,56^. Our study further revealed that these predicted compounds also showed binding with key catalytic residues, which proves that they can alter enzyme activity and reduce the viral burden. The selected drugs belong to antiviral and antibacterial families; therefore, they can be easily repurposed against ZIKV because they cannot inhibit the human targets and cause toxicity.

Cefpiramide is a well-known anti-bacterial compound active against *Pseudomonas aeruginosa*. It belongs to the semi-synthetic, broad-spectrum, beta-lactam, third-generation cephalosporin antibiotic family^63^. It inhibits bacterial cell wall synthesis by inactivating penicillin binding proteins (PBPs) through alteration of the final transpeptidation step, which is necessary for peptidoglycan cross-linking, a major cell wall component.^47,64^. Cefpiramide also showed antiviral activity against SARS-CoV-2. An in-silico study showed that Cefpiramide can inhibit the spike protein of SARS-CoV-2 with good binding affinity^65^. In another study, Cefpiramide was tested against the ACE2 receptor of SARS-CoV-2 and was observed to inhibit the receptor with −9.1 kcal/mol binding affinity^66^. In the current study, Cefpiramide showed both good binding affinity and stability in the MDS analysis, which indicates that it can act as a potential anti-ZIKV compound.

Ol_Me, also known as Benicar, is used to treat the high blood pressure (hypertension). It is an Angiotensin II Receptor Blocker^67^. It is a prodrug which is hydrolysed into Olmesartan during absorption from the gastrointestinal tract^68^. Although it is not known for its antiviral or antibacterial activity, in our study we found Ol_Me to be a potential NS5 inhibitor in the docking, binding free-energy and other MDS analyses. However, this drug has been shown to directly interact with human targets so minor changes would be required in this drug to propose it as an anti-ZIKV compound. In the future, researchers can test the efficacy of this drug against ZIKV NS5.

The overall analysis revealed that drug repurposing is a very powerful approach that can predict which FDA-approved drugs can be directly repurposed for diseases other than those for which they were originally intended.

## Conclusions

ZIKV is an arthropod-borne virus (arbovirus) belonging to the family of Flaviviridae and genus Flavivirus. As a single-stranded positive RNA virus, the genome of ZIKV is approximately 10 kb that encodes for three structural proteins and seven non-structural proteins. Inhibiting the proteins that are crucial for viral activity can reduce the growth of ZIKV. In this study, we used the available crystal structure of the NS5 protein and screened FDA-approved compounds to search for a potential inhibitor. A total of 2866 FDA-approved drugs were screened using virtual screening methods and the top five drugs were selected for evaluation of their ability to bind to NS5 using 150 ns MDS studies. Based on various MDS parameters such as RMSD, RMSF, Rg, SASA, PCA, and binding free energy, we found that, out of the five selected hits, Cefpiramide and Ol_Me formed stable interactions with NS5. Since the selected molecules are FDA-approved drugs, they may have an advantage in terms of their previous pharmacodynamics and pharmacokinetics properties. After evaluation, we propose that these may act as lead compounds for the development of potential inhibitors of NS5.

## Supplementary Information

Supplementary Table 1.

Supplementary Table 2.

## Data Availability

All data generated or analyzed during this study are included in this published article (and its Supplementary Information files).
